# Optimizing Parathyroid Preservation in Thyroidectomy: The Burjeel Protocol Utilizing Intraoperative Indocyanine Green Near-Infrared Fluorescence Imaging †

**DOI:** 10.3390/biomedicines13051044

**Published:** 2025-04-25

**Authors:** Iyad Hassan, Lina Hassan, Mohamad Askar, Rawan Khalid Salih

**Affiliations:** Department of Surgery, Burjeel Hospital, Abu Dhabi 7400, United Arab Emirates; lapsim1970@yahoo.de (L.H.); mohamad.askar@burjeel.com (M.A.); basedow1970@gmail.com (R.K.S.)

**Keywords:** fluorescence, hypoparathyroidism, indocyanine green, near-infrared imaging, parathyroid gland, postoperative hypocalcemia, surgical score, thyroid surgery

## Abstract

**Background**: Autofluorescence can identify parathyroid glands and protect their vasculature during thyroid surgery to prevent postoperative hypoparathyroidism. This study evaluates the Burjeel intraoperative protocol using near-infrared indocyanine green (ICG-NiR) imaging to preserve parathyroid glands during total thyroidectomy. **Methods**: This study conducted a single-centre retrospective matched cohort analysis involving 156 consecutive patients who underwent thyroidectomy using the Burjeel ICG-guided near-infrared (NiR) fluorescence protocol (“ICG group”). Patients were matched 1:1 based on gender and extent of resection with 156 counterparts who underwent standard thyroid surgery. The Stryker Spy-phi NiR fluorescence imaging system (Stryker™, Portage, MI, USA) was utilized in three modes—green, black/white, and colourful—to facilitate real-time visualization. Post-lobectomy, each parathyroid gland was individually scored for viability before the closure of the surgical site. Patients were stratified into hypoparathyroidism and euparathyroidism groups based on the parathyroid hormone levels measured on the first postoperative day. **Results**: The groups had 133 women and 23 men. Preoperative factors like age (43.7 years in both groups); resection time (49 min in the ICG group versus 50 min in the conventional group); and PTH, TPO, and Vit D3 levels were not statistically different. The ICG group had a lower rate of inadvertent parathyroidectomy (9% vs. 17.9% in the standard group, chi-square test, *p* = 0.015), a lower rate of postoperative hypoparathyroidism (18.6% vs. 35.3%, chi-square test, *p* = 0.001), and higher postoperative PTH levels (*t*-test, *p* = 0.0001). Postoperative hypoparathyroidism was associated with malignant surgical pathology and malignancy on both sides (*p* = 0.026 and 0.01, respectively). This study found that female participants had a higher incidence of unintentional parathyroidectomy (*p* = 0.001) but not postoperative hypoparathyroidism. Subgroup analysis showed a negative connection between ICG score and female hypoparathyroidism. **Conclusions**: The new Burjeel ICG-guided NiR fluorescence approach has greatly reduced inadvertent parathyroidectomy and hypoparathyroidism in female total thyroidectomy patients. Further research is needed to identify numerical variables that aid intraoperative decision-making.

## 1. Introduction

Thyroidectomy, a common surgical procedure for thyroid disorders, is associated with a risk of postoperative complications, particularly those related to the parathyroid glands. Temporary postoperative hypoparathyroidism is a serious complication that affects up to 46.8% of patients after total thyroidectomy (TT). Permanent hypoparathyroidism, although less common, affects up to 12.6% of patients with the condition [1,2]. This complication remains multifactorial and arises mainly from inadvertent damage to the gland’s blood supply and/or the unintentional removal of the parathyroid glands, leading to a deficiency in parathyroid hormone (PTH) secretion [3,4]. The incidence of inadvertent parathyroidectomy during thyroid surgery varies between 1% and 31% across various facilities [5,6,7,8]. However, postoperative hypoparathyroidism can cause a whole range of symptoms, from mild perioral tingling and numbness to severe cramps and carpopedal spasms. These symptoms can be very debilitating, preventing patients from being able to return to work, and in very severe cases, they can make self-care impossible. This can also have a knock-on effect, resulting in depression, anxiety, and loss of independence. Nevertheless, the clinical manifestation of hypoparathyroidism accounts for more than half of all readmission cases after cervical endocrine surgery [2]. Therefore, understanding the underlying mechanisms driving this condition is crucial for optimizing surgical techniques, refining patient management strategies, and enhancing outcomes in thyroid surgery. In recent years, intraoperative ICG-guided near-infrared imaging techniques have emerged as valuable tools to enhance surgical precision and reduce complications, including thyroid surgery. Among these techniques, indocyanine green near-infrared (ICG NIR) fluorescence imaging has garnered attention for its ability to provide real-time visualization of parathyroid glands during thyroidectomy. By leveraging the fluorescent properties of ICG, surgeons can accurately identify and preserve parathyroid tissue, thereby minimizing the risk of inadvertent injury or removal, and decide on possible auto-transplantation of the parathyroid gland at risk [9,10,11]. The potential advantages of ICG-NIRF are well recognized, and its implementation in various sectors is progressing rapidly. Nevertheless, numerous disparities among the utilized devices exist; the intraoperative imaging setting (such as the proximity of the NIRF camera to the imaging site, the presence of nearby illumination, the timing of the injection, and, notably, the amount of ICG employed and how it is diluted) remains a subject of ongoing discussion [12,13,14,15].

Vidal Fortuny and colleagues [16] previously published a study on the inaugural use of indocyanine green (ICG) to assess the blood flow of parathyroid glands during surgery. They investigated whether this technique might predict the function of the parathyroid glands following thyroidectomy and partial parathyroidectomy. These investigations showed that having one well-perfused parathyroid gland or a remnant of a parathyroid gland was enough to prevent hypoparathyroidism. Separate research documented comparable results in a cohort of 23 individuals who had thyroidectomy [17]. Nevertheless, a recent multicentric randomized controlled study did not demonstrate a decrease in the occurrence of transient hypoparathyroidism on the first day after surgery when using the ICG-Technology of the Fluobeam^®^ LX [18].

The identification of a dependable scoring system for the ICG fluorescence technique in parathyroid evaluation during thyroid surgery is of the utmost importance to surgeons and serves a number of critical functions. Firstly, it provides a standardized method for evaluating the intensity and uniformity of fluorescence in the parathyroid glands. This consistency allows surgeons to make reliable assessments of gland viability and function intraoperatively. Secondly, the scoring system helps surgeons differentiate between healthy parathyroid tissue and other structures, such as lymph nodes or thyroid tissue, that may exhibit fluorescence [19].

By distinguishing between these tissues, surgeons can confidently preserve functional parathyroid glands while removing surrounding thyroid tissue or lymph nodes. Additionally, the scoring system aids in decision-making during surgery. Surgeons can use the fluorescence intensity and uniformity scores to guide their actions, such as determining whether additional exploration is necessary to locate all parathyroid glands or whether further intervention, such as gland auto-transplantation, is warranted to prevent postoperative hypoparathyroidism [20].

This cross-matched retrospective study was conducted with the purpose of determining whether or not a specially defined protocol, called the Burjeel technique, which makes use of the Stryker Spy Phi Near Infrared imaging System (Stryker™, Portage, MI, USA) in conjunction with a special dilution of indocyanine green, has the potential to reduce the incidence of postoperative hypoparathyroidism and decrease the rate of inadvertent parathyroidectomy in a single thyroid surgery centre.

## 2. Materials and Methods

This study conducted a retrospective matched cohort analysis of 312 thyroidectomy patients treated between January 2022 and March 2024 in a tertiary-care private hospital in Abu Dhabi, United Arab Emirates. The study aimed to compare the early postoperative parathyroid function of ICG-NiR-guided thyroidectomy (“ICG-group”) using the Burjeel protocol, a novel ICG technique, with that of standard thyroidectomy (“ST-group”). The patients were paired in a 1:1 ratio based on their gender, age, and the extent of their thyroid resection. The surgeries were conducted only by a proficient endocrine surgeon (I.H.). To ensure the complete eradication of any potential selection bias, the operating room coordinator used digital logbooks to carry out a randomized selection of patients for the control group. All patients participating in the research provided informed consent. The exclusion criteria for the research were those with an ASA score greater than 3, pregnant women, those aged below 18 years, and those who declined to participate. The ICG score, duration of surgery, surgical histology, unintentional removal of parathyroid glands, postoperative parathyroid function test, and rate of clinical hypoparathyroidism were assessed in both groups. Data on demographic variables, preoperative investigations, and intra- and postoperative parameters were collected using a standard case record spreadsheet.

### 2.1. Burjeel ICG-NiR Autofluorescence Protocol

All thyroidectomy procedures were performed by experienced thyroid surgeons utilizing a standardized technique; see below. We then exposed the thyroid gland and identified the parathyroid glands. We diluted the ICG dye (Aurogreen, Aurolab^®^, Madurai, India) in 10 mL of G10% solution to take advantage of the fact that parathyroids are very metabolically active and have a large number of mitochondria. This is in line with the principles of molecular imaging, which uses glucose to visualize lesions. However, diluting ICG in glucose solution for the fluorescence imaging of the parathyroid glands offers several advantages, including enhanced signal intensity, reduced background noise, an improved safety profile, and enhanced clinical translation. This approach holds promise for improving the accuracy, reliability, and clinical utility of ICG fluorescence imaging in the evaluation and management of parathyroid disorders.

The SPY PHI imaging system (Stryker™, Portage, MI, USA software version LCB 4.32) operates at a single wavelength of 805 nm, and the difference seen on photos is a result of native images processed by a software algorithm. For the highest ICG concentration in a tissue, the colour appears red/orange, then blue, and if there is no ICG in the tissue, it appears grey. To avoid the wrong interpretation of the images, the difference between the modes and the distance of the camera to the parathyroid should remain stable at 15 cm.

**Figure 1:**
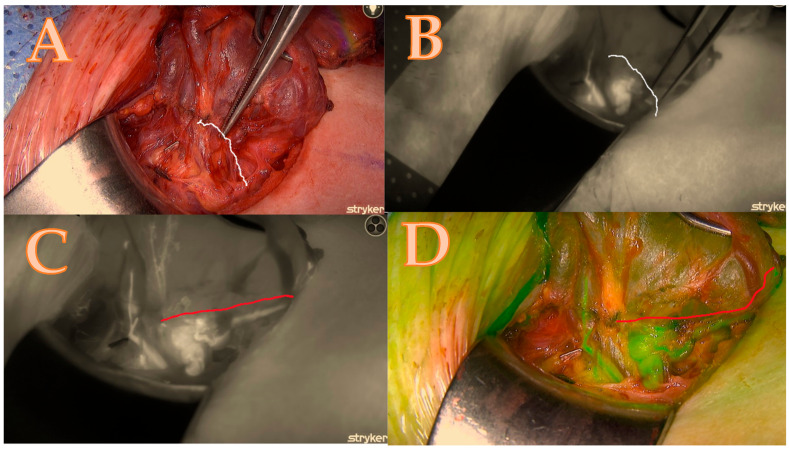
Right lower parathyroid; (**A**) is a native picture that represents the initial step of the Burjeel ICG technique, while the white line represents the theoretical dissection pathway using a classical technique without ICG. (**B**) The initial ICG brightness and vascular path of the black-and-white ICG illustration determine the parathyroid dissection pathway without an optimal and final ICG image. (**C**) = maximum brightness of the ICG illustration in black and white with additional information about the entire circle of blood supply origin that modified the parathyroid dissection pathway shown in the red line; (**D**) = image confirmation in green mode to minimize errors and contamination with the background.

After the thyroid lobe was mobilized in the wound, a 5 mL bolus of ICG-diluted solution (Aurogreen, Aurolab^®^, Madurai, India) (0.3 mg/kg) was injected intravenously. Simultaneously, the Stryker spy camera was adjusted to the black/white brightness mode and started recording after reducing the theatre light to obtain optimal fluorescence images. Approximately 90 s after the intravenous injection, the Stryker near-infrared fluorescence system began to detect the fluorescence signal given by the ICG. This allowed for the real-time identification of the blood arteries that feed the parathyroids, which are of primary importance and can change the strategy of dissection as seen in Figure 1A–D.

**Figure 2:**
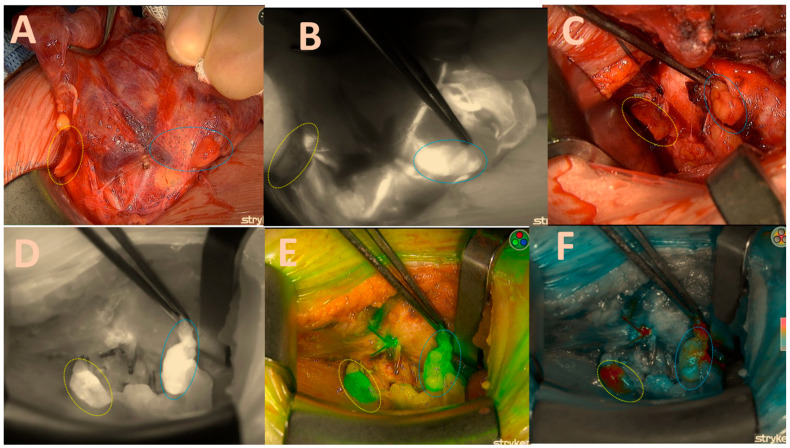
Both right-sided parathyroids; (**A**) = native image. The yellow circle depicts the upper parathyroid that remains attached to the thyroid capsule, while the blue circle signifies the lower parathyroid that has become lodged in the capsule. (**B**) = good brightness of the ICG black-and-white mode illustration of the lower parathyroid and its vascular pathways; (**C**) = native picture of both parathyroids after right lobectomy; (**D**) = excellent black/white brightness of the ICG illustration of the lower gland (2 points); moderate inhomogeneous brightness of the upper gland (1 point); (**E**) = good green mode of the upper gland (2 points); moderate inhomogeneous brightness of the lower gland (1 point). (**F**) = good colour brightness of the ICG depiction of the lower gland (2 points), moderate inhomogeneous brightness of the upper gland (1 point), total gland score of 11 of a maximal score of 14 displayed in Table 1.

Once a sufficient amount of ICG dye is delivered into the parathyroid gland, it becomes visible as a white fluorescent structure (Figure 2B).

**Figure 3:**
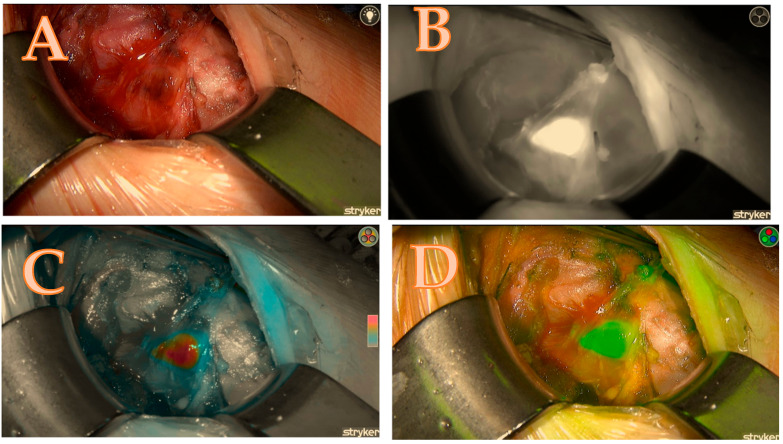
Right upper parathyroid; (**A**) = native image, (**B**) = the maximum brightness of the ICG illustration in black-and-white mode, (**C**) = the maximum brightness of the ICG illustration in coloured mode, (**D**) = the maximum brightness of the ICG illustration in green mode; a total score of 7/7.

The ability to alternate between the three fluorescence modes, namely black/white, green, and coloured mode, in the Spy-phi imaging system aids in accurately determining the actual intensity of ICG concentration retained within the gland. The homogeneity of the distribution is not only of significant relevance but the colour mode also specifically displays the true intensity from which the Burjeel score is derived, as follows: The colour grey indicates a lack of concentration of ICG in the gland, resulting in a score of zero. The colour blue indicates a moderate concentration of ICG, resulting in a score of one. The colour red or orange indicates an excellent concentration of ICG in the gland, resulting in a score of 2 (Figure 3C).

ICG Scoring Methodology: The ICG score was calculated based on the surgeon’s assessment of parathyroid viability during the thyroidectomy, incorporating both visual confirmation and the use of near-infrared (NiR) fluorescence imaging. Each parathyroid gland was evaluated using the Stryker Spy-phi NiR fluorescence system in three modes: green, black/white, and red/orange/blue/grey. A maximum score of 7 could be assigned to each parathyroid gland, based on the following criteria. Surgeon’s Assessment (Vitality): If the surgeon identified and confirmed the vitality of a parathyroid gland, it received an additional point in the scoring system, reinforcing the importance of clinical judgement in conjunction with imaging. Fluorescence Imaging (ICG Modes): Green Mode: A score of 2 points was awarded if the gland demonstrated strong fluorescence in the green mode, indicating good vitality. If the gland showed weaker fluorescence, it received 1 point. Black/White Mode: In this mode, 2 points were given if the gland’s boundaries were well defined. A lower score of 1 point was awarded for weak or ambiguous visualization. Red or Orange/Blue/Grey Mode: A score of 2 points was assigned for an orange-coloured parathyroid gland, 1 point for blue, and 0 for grey fluorescence. Total Score: The total score for each gland was calculated by adding the points from the surgeon’s assessment and fluorescence imaging. A gland could receive a maximum of 7 points, broken down as follows:

▪ Green: 2 points for strong fluorescence;
▪ Black/White: 2 points for clear visualization;
▪ Red/Orange/Blue/Grey: 2 points for visible identification;
▪ Vitality confirmation by the surgeon: 1 point.

Example calculation: for the right upper parathyroid gland,

- Green mode: 2 points;
- Black/White mode: 1 point;
- Red/Orange/Blue/Grey mode: 2 points;
- Surgeon’s assessment (vitality confirmation): 1 point;
- Total score: 6 points out of a possible 7.

**Figure 4:**
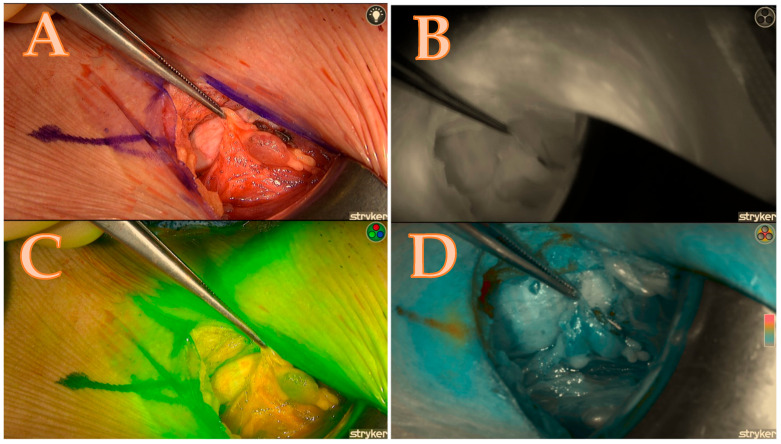
Right upper parathyroid; (**A**) = native image, (**B**) = the maximum brightness of the ICG illustration in black-and-white mode, (**C**) = the maximum brightness of the ICG illustration in coloured mode, (**D**) = the maximum brightness of the ICG illustration in green mode; a total score of 4/7.

Overall Scoring:

- The total score for each gland was tallied, and the aggregate score for the entire set of glands was calculated. For example, in a case where both right upper and right lower glands scored 6 and 5 points, respectively, the total score for both glands would be 11 out of a possible 14 points.
- The score ratio is calculated by dividing the total score by the maximum possible score and converting it into a percentage. In this case, 11/14 = 78.5%. This methodology allows for a comprehensive and standardized assessment of parathyroid gland viability, combining clinical assessment with advanced fluorescence imaging (see Table 1 and Figure 1, Figure 2, Figure 3 and Figure 4).

### 2.2. Surgical Technique

We have previously described the standard operating room (OR) procedures for thyroid surgery at our institution, including patient placement on the OR table, anesthesia selection, equipment setup, and neuromonitoring [20]. In summary, a minimally invasive open thyroidectomy was performed under general anesthesia without neuromuscular blocking, facilitating intraoperative neuromonitoring. Key steps included patient positioning, electrode placement (ECG, pulse oximeter, blood pressure monitor), and baseline vital sign documentation. After pre-oxygenation, midazolam, lidocaine, propofol, and remifentanil were administered, followed by intubation using video laryngoscopy and the placement of an electromyogram (EMG) endotracheal tube.

A 2.5–4 cm Kocher incision was made, and the strap muscles were retracted to visualize the inferior thyroid artery (ITA) and middle thyroid vein. The vagal nerve (VN) was identified within the carotid sheath, and neuromonitoring was employed to rule out non-recurrent laryngeal nerve (RLN)’s presence. The RLN signal was tested using 1 mA stimulation and a 200 µV EMG response.

Bipolar cautery and LigaSure^®^ (Medtronic, Minneapolis, MN, USA) were used for vein and muscle retraction, and the thyroid lobe was carefully extracted. The superior laryngeal nerve (SLN) was protected, and upper pole vessels were ligated after EMG testing. The procedure preserved parathyroid function by maintaining blood supply to all parathyroid glands.

### 2.3. Postoperative Parathormone Monitoring

On the first postoperative day, serum parathyroid hormone level (PTH) was systematically measured in pmol/L in all patients. The biomarker was selected as a primary indicator for evaluating early parathyroid function and detecting transient hypoparathyroidism. PTH was measured using a standardized chemiluminescent immunoassay (Elecsys PTH, Roche Diagnostics, Mannheim, Germany). The data were used to classify patients into either the euparathyroid or hypoparathyroid group for further analysis.

### 2.4. Statistical Analysis

The statistical analysis was conducted using IBM SPSS version 29 (SPSS Inc., Chicago, IL, USA). The parametric data are shown as the average value together with the measure of variability, which is the standard deviation. On the other hand, the non-parametric data are represented by the middle value (median rank) along with the range between the first and third quartiles (interquartile range). The univariate analysis included Student’s *t*-tests and Mann–Whitney U-tests for continuous variables and Fisher’s exact test for categorical data. A *p*-value was considered statistically significant if it was below 0.05.

## 3. Results

*Demographic Characteristics*

The study population consisted of 312 patients, with 156 in the ICG group and 156 in the standard group. Both groups were matched 1:1 by gender and extent of resection. Each group comprised 133 females (85.3%) and 23 males (14.7%). The mean age was 43.7 ± 9.2 years in the ICG group and 43.6 ± 9.4 years in the standard group (*p* = 0.89). There were no statistically significant differences in preoperative serum PTH, TPO antibodies, or vitamin D3 levels between the groups, confirming baseline comparability.

**Figure 5:**
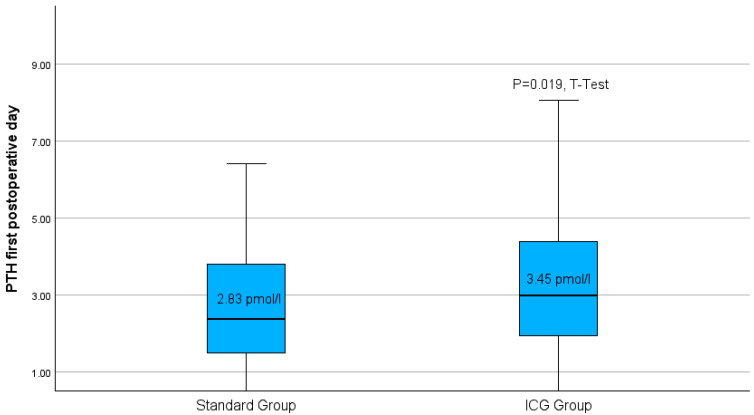
Boxplots of parathyroid hormone level on the first postoperative day for both study groups. *p* = 0.019 in the *t*-test.

Following thyroidectomy, the group treated with the Burjeel intraoperative ICG-protocol had a notably greater average of parathormone measurement compared to the standard group (Figure 5).

In addition, the incidence of inadvertent parathyroidectomy as well as transient postoperative hypoparathyroidism (defined as PTH < 1.5 pmol/L) was reduced in the ICG group compared to the conventional group (Table 2).

**Figure 6:**
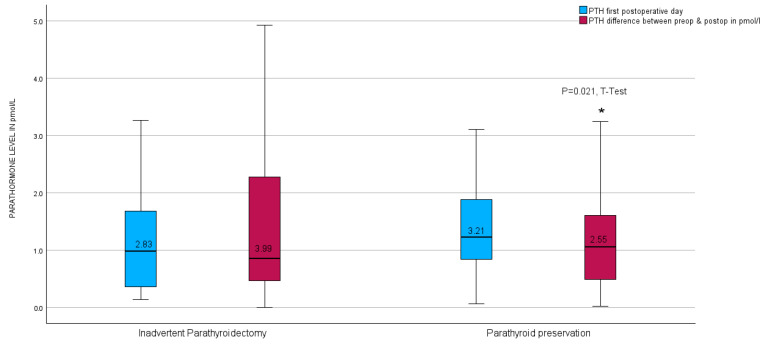
Boxplots of parathyroid hormone level calculated difference between preoperative and first postoperative day level after thyroid resection in relation to inadvertent parathyroidectomy in the entire group. * red boxplots indicate a *p* = 0.021 in the *t*-test.

Upon further investigation, no notable disparity was observed in the parathyroid hormone (PTH) levels on the initial postoperative day between the unintended parathyroidectomy group and the group lacking inadvertent parathyroid gland presence in the surgical specimen. Nevertheless, a significant contrast emerged in the calculated difference in PTH levels measured before and after the surgical procedure (PTH-Delta), as illustrated in Figure 6.

Nevertheless, postoperative hypoparathyroidism was observed in 42.9% of the 42 patients who experienced an inadvertent parathyroidectomy, compared to 24.5% of the 269 patients whose parathyroids were preserved during surgery. According to Fisher’s exact test, the difference in the hypoparathyroidism rate between the two groups was statistically significant (*p* = 0.013). Furthermore, there was a significant correlation between postoperative hypoparathyroidism and malignant surgical pathology as well as the presence of malignancy on both sides (*p* = 0.026 and *p* = 0.01, respectively). Among all participants of this study, being female was associated with a greater risk of inadvertent parathyroidectomy (*p* = 0.001) but not with postoperative hypoparathyroidism. Subgroup analysis revealed a negative correlation between the ICG score and the occurrence of hypoparathyroidism in the female gender.

## 4. Discussion

Transient hypoparathyroidism is a common consequence following thyroid surgery, impacting up to 60% of patients [17,21,22]. Even highly skilled surgeons may unintentionally remove or disrupt the blood supply to the parathyroid glands while performing thyroid surgery, since these glands are tiny and have a similar colour to fatty tissue. The etiology of post-thyroidectomy hypoparathyroidism is multifaceted, with factors such as autoimmune disease, malignancy, prior radiation, radioactive iodine, Magnesium and Vit-D deficiency, and type of anesthesia being identified as potential contributors [23,24,25]. Therefore, it is crucial to accurately identify and protect the parathyroid glands during thyroidectomy in order to minimize the likelihood of developing postoperative hypocalcemia. Previously, the viability of parathyroids during thyroidectomy was assessed based on subjective criteria and the surgeon’s expertise. Near-infrared autofluorescence, in conjunction with indocyanine green (ICG) dye, constitutes a pioneering technique utilized in thyroid surgery for the precise localization and preservation of parathyroid glands. Its adoption is increasingly prevalent within the realm of endocrine surgery. However, the controversy surrounding the use of this novel technology to enhance the detection of parathyroid glands, as opposed to the traditional approach, persists. Priyanca et al. did not see an elevated rate of detected parathyroid glands while using the ICG imaging equipment; however, they did find a reduced incidence of postoperative hypoparathyroidism compared to the usual technique [26]. However, other research groups have observed a higher detection rate of parathyroid glands using ICG-fluorescence technology compared to the usual technique [27]. Although we did not gather data for the control group on the number of identified parathyroid glands, the lower rate of unintentionally identified parathyroid glands found on final histopathology in the ICG group compared to the standard group suggests that ICG may have improved our ability to identify more parathyroid glands.

Another point of contention revolves around whether this new approach effectively reduces postoperative hypoparathyroidism. The majority of studies, including ours, which utilized a historical control group, demonstrate a decrease in the incidence of postoperative hypoparathyroidism [28,29,30,31,32,33]. Additionally, some monocentric randomized controlled trials have reported a reduction in postoperative hypoparathyroidism through the use of ICG-near-infrared technology compared to control groups [34,35]. However, a larger multicentric randomized trial has yielded mixed results. While they did not show a reduction in the overall rate of postoperative hypoparathyroidism, subgroup analyses revealed fewer instances of postoperative hypoparathyroidism and increased the identification of parathyroid glands in patients undergoing neck dissection using this technique compared to control groups [36]. The primary results of our study showed that patients who had thyroid resection with the assistance of the Burjeel protocol for intraoperative ICG-near-infrared technology had greater levels of parathyroid hormone on the first day after surgery compared to the standard group. Furthermore, the incidence of the unintentional removal of the parathyroid gland identified in the definitive surgical pathology, as well as the occurrence of clinical hypoparathyroidism, was notably reduced in the ICG-NiR group.

Currently, there is insufficient information to establish an unequivocal relationship between the severity of parathyroid gland devascularization and the occurrence of postoperative hypoparathyroidism. Nevertheless, several studies have shown that intraoperative ICG angiography may promptly judge parathyroid function after thyroid resection, providing a benefit over traditional approaches such as intraoperative parathormone measurement. This has led to an upsurge in research into developing an ICG score system during thyroid surgeries [37]. Vidal Fortuny et al. scored PGs by ICG fluorescence, rating glands as 0, 1, or 2 for vascularization. Their approach gave non-vascularized glands 0 points and well-vascularized glands 2 points. He concluded that an ICG score of 2 in at least one PG reflected normal postoperative PTH levels [38]. Rudin et al. evaluated 86 ICG-angiography-guided total thyroidectomy patients. Unlike Vidal Fortuny et al., they observed that at least two glands with an ICG score of 2 corresponded with normal PTH levels [39]. In our study, the progressive reduction in hypoparathyroidism could suggest that these modifications in the surgical technique occurred gradually, after the surgeons were confident with the results suggested by the Burjeel ICG-near-infrared scoring system. Another possible explanation is that ICG angiography might increase the awareness of complications involving parathyroid gland preservation (the Hawthorne effect) [39,40]. The surgeon may have been alerted to the possibility of evaluating every parathyroid gland with ICG angiography and would thus subconsciously focus on a more meticulous dissection of the vascular pedicle. The potential influence of the Hawthorne effect was mitigated to some extent by the standardization of intraoperative procedures. All operations were performed by a single experienced endocrine surgeon using a well-established protocol, with the Burjeel ICG-NIR technique integrated into routine practice. This consistency in surgical approach reduces performance variability and limits observer-driven behaviour change. While it is acknowledged that the Hawthorne effect cannot be entirely eliminated, the uniform application of the protocol enhances the reliability of the observed relationship between intraoperative imaging and postoperative outcomes.

However, in our study, the Burjeel ICG-near-infrared score correlated with inadvertent parathyroidectomy and early postoperative hypoparathyroidism.

Finally, in our study, female patients showed a higher incidence of inadvertent parathyroidectomy without a corresponding increase in postoperative hypoparathyroidism. Several factors likely contributed to this finding. Female parathyroid glands, often smaller and located in delicate tissue planes, may be more vulnerable to unintentional removal during surgery. Additionally, estrogen’s influence post-surgery could impact calcium regulation and facilitate parathyroid hormone recovery, potentially mitigating hypoparathyroidism even if glands are inadvertently removed.

Moreover, our findings highlight a robust association between postoperative hypoparathyroidism and malignant surgical pathology, especially in cases of bilateral malignancy. In such high-risk scenarios, the meticulous preservation of parathyroid glands is critical. ICG fluorescence imaging emerges as a preventive strategy, particularly beneficial for female patients with bilateral multifocal malignancy.

Our study has several limitations. Its retrospective, single-centre design limits generalizability and may introduce selection bias. Moreover, matching only by gender and extent of resection could have overlooked other important confounding factors. The relatively small sample size, dependence on the specific Stryker Spy-phi NiR imaging system, and the use of an unvalidated parathyroid scoring system may affect both the reproducibility and broader accessibility of the technique. Finally, long-term outcomes such as recurrence and sustained parathyroid function were not assessed.

In conclusion, the Burjeel ICG-NIR fluorescence protocol for parathyroid preservation in thyroidectomy significantly reduced inadvertent parathyroidectomy and postoperative hypoparathyroidism, as demonstrated by higher early postoperative PTH levels and a lower incidence of gland removal compared to the standard approach. Our findings suggest that this novel protocol—employing a specific ICG dilution and a tailored scoring system using the Stryker Spy-phi imaging system—can optimize parathyroid preservation without compromising surgical radicality. Although our study is limited by its retrospective, single-centre design, small sample size, and reliance on an unvalidated scoring system, the promising results warrant further prospective research to refine numerical criteria for intraoperative decision-making and to assess long-term outcomes.

## Figures and Tables

**Table 1:** The calculated score of the Burjeel ICG scoring technique, which corresponds to Figure 4. NA means not applicable.

	Right Upper Parathyroid	Right Lower Parathyroid	Left Upper Parathyroid	Left Lower Parathyroid	Total Score	Score Ratio
Number of Glands	1	1	NA	NA	2	2/2
Green	2	1	NA	NA	3	3/4
Black/White	1	2	NA	NA	3	3/4
Red or Orange/Blue/Grey	2	1	NA	NA	3	3/4
Total Score	6	5	NA	NA	11	11/14 = 78.5%

**Table 2:** Postoperative parathyroid function data of both study groups. *p*-values serve as an indicator of statistical significance in Fisher’s exact test.

	Standard Group	ICG-Group	*p*-Value
Postoperative Euparathyroidism (n)	101/156 (64.7%)	127/156 (81.4%)	
Postoperative Hypoparathyroidism (n)	55/156 (35.3%)	29/156 (18.6%)	0.001
Histopathology with Inadvertent Parathyroidectomy (n)	28/156 (17.9%)	14/156 (9%)	0.015
Histopathology Without Inadvertent Parathyroidectomy (n)	128/156 (82.1%)	142/156 (91%)	
Mean Surgery Duration in Minutes (min)	49.61 min	49.30 min	

## Data Availability

The data that support the results of this study can be requested from the corresponding author.

## Abbreviations

List of the abbreviations used in the manuscript:

ICG	Indocyanine Green
NiR	Near-Infrared
PTH	Parathyroid Hormone
TPO	Thyroid Peroxidase
Vit D3	Vitamin D3
ASA	American Society of Anesthesiologists
EMG	Electromyogram
SLN	Superior Laryngeal Nerve
RLN	Recurrent Laryngeal Nerve
SPSS	Statistical Package for the Social Sciences
CT	Computed Tomography
BMI	Body Mass Index
